# Vanin-1 in Renal Pelvic Urine Reflects Kidney Injury in a Rat Model of Hydronephrosis

**DOI:** 10.3390/ijms19103186

**Published:** 2018-10-16

**Authors:** Keiko Hosohata, Denan Jin, Shinji Takai, Kazunori Iwanaga

**Affiliations:** 1 Education and Research Center for Clinical Pharmacy, Osaka University of Pharmaceutical Sciences, Osaka 569-1094, Japan; iwanaga@gly.oups.ac.jp; 2 Department of Innovative Medicine, Osaka Medical College, Osaka 569-8686, Japan; pha012@osaka-med.ac.jp (D.J.); pha010@osaka-med.ac.jp (S.T.)

**Keywords:** hydronephrosis, renal tubular damage, early biomarker, renal pelvic urine, voided urine, epithelial-to-mesenchymal transition

## Abstract

Urinary tract obstruction and the subsequent development of hydronephrosis can cause kidney injuries, which results in chronic kidney disease. Although it is important to detect kidney injuries at an early stage, new biomarkers of hydronephrosis have not been identified. In this study, we examined whether vanin-1 could be a potential biomarker for hydronephrosis. Male Sprague-Dawley rats were subjected to unilateral ureteral obstruction (UUO). On day 7 after UUO, when the histopathological renal tubular injuries became obvious, the vanin-1 level in the renal pelvic urine was significantly higher than that in voided urine from sham-operated rats. Furthermore, vanin-1 remained at the same level until day 14. There was no significant difference in the serum vanin-1 level between sham-operated rats and rats with UUO. In the kidney tissue, the mRNA and protein expressions of vanin-1 significantly decreased, whereas there was increased expression of transforming growth factor (TGF)-β1 and Snail-1, which plays a pivotal role in the pathogenesis of renal fibrosis via epithelial-to-mesenchymal transition (EMT). These results suggest that vanin-1 in the renal pelvic urine is released from the renal tubular cells of UUO rats and reflects renal tubular injuries at an early stage. Urinary vanin-1 may serve as a candidate biomarker of renal tubular injury due to hydronephrosis.

## 1. Introduction

Ureteral obstruction is a common cause of kidney damage and insufficiency. As a result of mechanical hindrance caused by kidney stones, malignancy and benign prostatic hyperplasia [1], hydronephrosis may develop in kidneys. Unlike other kidney diseases, obstructive nephropathy is often reversible with early diagnosis and treatment. However, without timely and correct treatment, it may lead to renal failure. Therefore, it is important to detect kidney injuries at an early stage of hydronephrosis induced by renal obstruction. Currently, the most commonly used examinations for the assessment of renal obstruction are diuretic dynamic renograms using radiopharmaceuticals but they cannot detect kidney injury early enough to ensure optimal treatment.

We previously found that the urinary excretion of vanin-1 is elevated before the increase in conventional renal biomarkers in rats with nephrotoxicant- or drug-induced renal tubular injury [2,3]. Urinary vanin-1 is a potential novel biomarker for acute kidney injuries (AKI), and furthermore, we have investigated its reliability in diagnosing AKI compared with other established markers [4]. The vanin-1 molecule contains a C-terminus consensus sequence that couples a glycosylphosphatidylinositol (GPI) moiety, which is anchored at the membrane [5,6], and it is a pantetheinase that catalyzes the conversion of pantetheine into pantothenic acid (vitamin B5) and cysteamine. Vanin-1 was found in rats with renal ischemia–reperfusion injuries, which form a model of renal injury caused by oxidative stress [7]. In addition, it has been reported that vanin-1 is a tissue sensor for oxidative stress and it acts as a regulator of the tissue response to oxidative stress [8]. Recently, vanin-1 was reported to be found in the urine of animals with severe renal inflammation and fibrosis induced by titanium dioxide nanoparticles [9].

In rodents, unilateral ureteral obstruction (UUO) is a well-known model in which urine accumulates in the renal pelvis, leading to hydronephrosis with renal atrophy and interstitial fibrosis [10]. This process involves the activation of the renin-angiotensin system [11], expression of transforming growth factor-β1 (TGF-β1) [12] and the induction of tubular cell apoptosis [13]. In addition, oxidative stress has been identified as an important pathogenic mechanism in the development of renal interstitial fibrosis in UUO [14]. Vanin-1 is associated with oxidative stress, and therefore, it is possible that urinary vanin-1 levels are also associated with hydronephrosis. In this present study, we investigated whether urinary vanin-1 was present in urine from the renal pelvis of rats with UUO.

## 2. Results

### 2.1. Echographic and Histological Changes in the Kidneys of Rats with UUO

**Figure 1:**
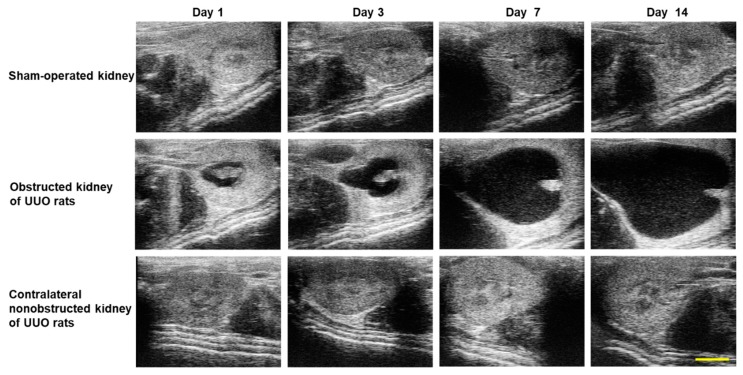
Echographic images of kidneys from sham-operated rats and rats with UUO. Representative echographic images of kidneys from sham-operated rats and rats with UUO using the Vevo 1100 Imaging System. Scale bar: 5 mm.

The echographic images of kidneys obtained from sham-operated rats and rats with UUO are shown in Figure 1. Depending on the ligation time, the urine increased in the renal pelvis of the obstructed kidney of rats with UUO. There was no difference between the sham-operated kidney and contralateral non-obstructed kidney.

**Figure 2:**
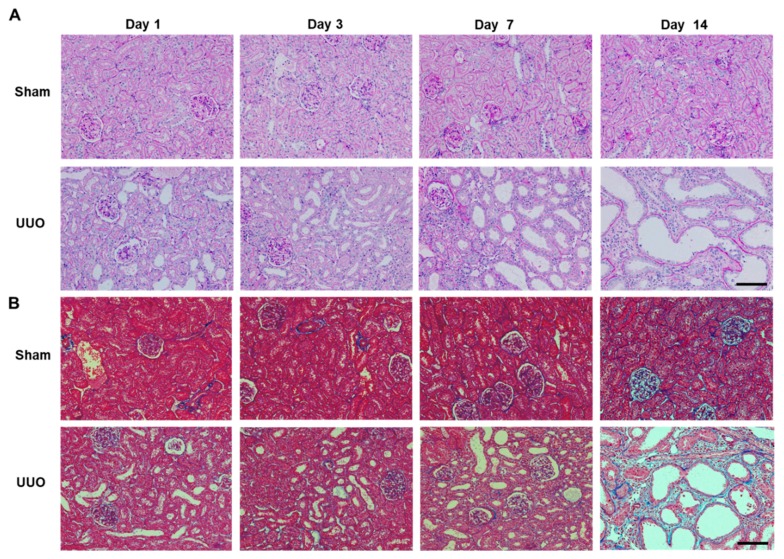
Histological images of obstructed kidneys of rats with UUO. (**A**) Periodic acid-Schiff (PAS) staining of sham-operated kidney (Sham) and obstructed kidney (UUO). There was obvious renal tubular damage on days 7 and 14. Scale bar: 100 μm. (**B**) Masson’s trichrome staining microphotographs of sham-operated kidney (Sham) and obstructed kidney (UUO). Focal fibrotic changes on day 7 became severe on day 14. Scale bar: 100 μm.

In addition to the structural deformities in the obstructed kidneys of rats with UUO, histological changes, such as tubular dilatation, tubular atrophy and a widened interstitial space, were observed (Figure 2A). In contralateral non-obstructed kidneys of rats with UUO, there were no histological changes after the development of secondary hydronephrosis. Masson’s trichrome staining revealed only limited renal interstitial fibrosis on day 7 after UUO, whereas fibrosis was more extensive on day 14 after UUO (Figure 2B).

### 2.2. Vanin-1 Was Released in Renal Pelvic Urine

**Figure 3:**
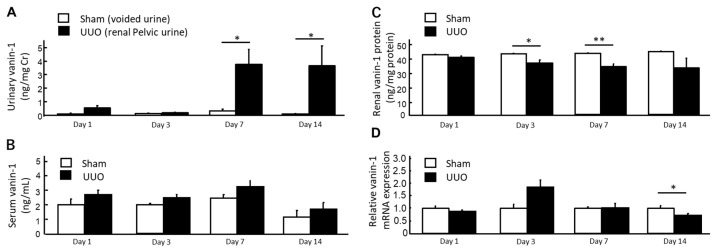
Accumulation of vanin-1 in the renal pelvic urine of rats with UUO. (**A**,**B**) Released vanin-1 was detected in the renal pelvic urine, but not in the serum, on days 7 and 14 after UUO (*n* = 5 for each time point in each group). Data represent the mean + SE. * *p* < 0.05 vs. the sham-operated rats (by one-way ANOVA followed by Tukey’s post hoc analysis). (**C**,**D**) Vanin-1 protein and mRNA expressions in the renal cortices of the obstructed kidney (UUO, *n* = 5 per time point) were decreased compared with those of sham-operated kidney (Sham, *n* = 5 per time point). Data represent the mean + SE. * *p* < 0.05 and ** *p* < 0.01 vs. the sham-operated rats (by one-way ANOVA followed by Tukey’s post hoc analysis).

In the rats with UUO, as the voided urine originated from the contralateral non-obstructed kidneys, we used renal pelvic urine for urinalysis. By contrast, in the sham-operated rats, it was not possible to collect renal pelvic urine and therefore, we used voided urine for urinalysis. As shown in Figure 3A, the urinary vanin-1 levels in the rats with UUO were significantly higher than those in the sham-operated rats on days 7 and 14.

There were no significant differences in serum vanin-1 levels between the groups. No significant difference in the renal vanin-1 protein expression between the UUO and sham-operated groups was noted, but there was a significant decrease in the renal vanin-1 protein expression in the rats with UUO on days 3 and 7 after UUO.

**Figure 4:**
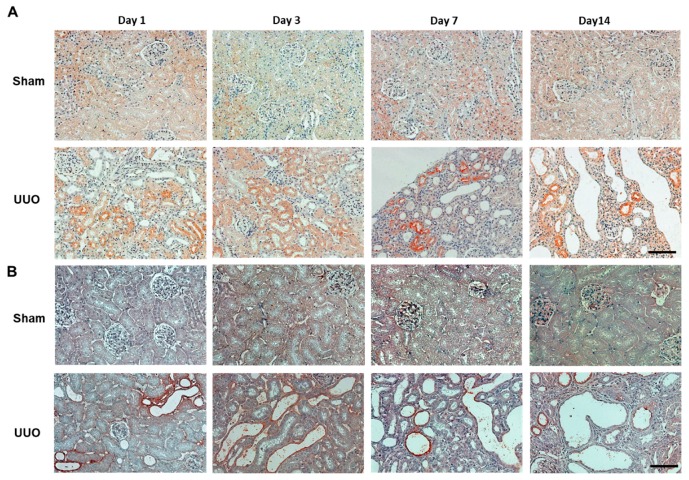
Representative of immunostaining of vanin-1 and 4-HNE in the obstructed kidney of rats with UUO. (**A**) Vanin-1 expression was localized both around the injured tubules and within a subset of injured tubules, but not in the area with interstitial fibrosis. (**B**) 4-HNE, which is an oxidative stress marker, was positive in the renal tubules of obstructed kidney of rats with UUO. Scale bar: 100 μm.

The immunohistochemical staining of vanin-1 in the renal cortices is shown in Figure 4. In the sham-operated rats, vanin-1 was slightly and extensively expressed. In the rats with UUO, vanin-1-positive staining was more intense not only around the injured tubules, but also within a subset of injured tubules (Figure 4A), but its expression was observed in areas where the staining of 4-HNE, which is an oxidative stress marker, was positive (Figure 4B) but not in the interstitial fibrotic area.

### 2.3. Involvement of the EMT in Progression to Interstitial Fibrosis of Rats with UUO

**Figure 5:**
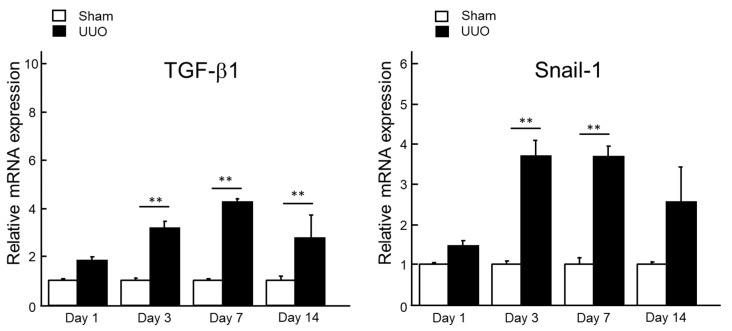
The mRNA expression levels of TGF-β1 and Snail-1 in the renal cortices after UUO. Total RNA was extracted from renal cortices from sham-operated kidney (Sham, *n* = 5 per time point) and obstructed kidney (UUO, *n* = 5 per time point), and the relative mRNA expression levels of TGF-β1 and Snail-1 were measured by real-time PCR. The mean value of the sham-operated rats was set to 1 and the data represent the mean + SE. **p < 0.01 vs. the sham-operated rats (by one-way ANOVA followed by Tukey’s post hoc analysis).

As vanin-1 is expressed in renal epithelial cells, we suspected that vanin-1 expression will decrease due to early epithelial–mesenchymal transition (EMT) and subsequent fibrosis. Thus, we examined Snail-1, a zinc finger transcription factor, which has been reported to repress the transcription of E-cadherin by binding to the E box elements in the E-cadherin promoter. Thus, this induces EMT [15] and TGF-β1 expression. TGF-β1 plays a major role in the induction of EMT and renal fibrosis [16], exerting its action through apoptosis, increased synthesis and decreased degeneration of the extracellular matrix as well as induction of EMT. As shown in Figure 5, TGF-β1 mRNA expression in renal cortices was significantly increased on days 3, 7 and 14 after UUO, which is consistent with the progression of hydronephrosis. Snail1 mRNA expression in renal cortices was also significantly upregulated on days 3 and 7 after UUO.

## 3. Discussion

The present study revealed that released vanin-1 was present in renal pelvic urine from rats with UUO, while there was only a small amount in the voided urine from sham-operated rats. In the UUO model, as the left ureter was ligated at two points, voided urine originated from the right reference kidney. Therefore, the presence of vanin-1 in the renal pelvic urine may reflect renal tubular injury due to hydronephrosis. Considering that obstructive nephropathy in humans is always due to incomplete obstruction, our findings suggest that urinary vanin-1 may be a useful biomarker for hydronephrosis.

Vanin-1 is a key molecule for regulating the glutathione (GSH)-dependent response to oxidative injury in epithelial cells [8,17]. Vanin-1 increases cellular stress by generating cysteamine, which itself suppresses gamma-glutamylcysteine synthetase (**γ**GCS), a rate-limiting enzyme for GSH synthesis [8], and decreases reactive oxygen species (ROS)-detoxifying enzymes, such as superoxide dismutase and glutathione peroxidase [18]. In the present study, the positive and intense 4-HNE (an oxidative stress marker) expression was observed in the renal proximal tubules on days 1, 3 and 7 after UUO. It is important to note that this was followed by an expression of vanin-1 in the injured renal tubular epithelial cells of obstructed kidneys (Figure 4). As reported by several studies, ROS mediate the profibrotic action of TGF-β1, which mainly functions in the induction of renal fibrosis via EMT [16,19,20,21]. Our study revealed that TGF-β1 was significantly upregulated in a time-dependent manner on days 3, 7 and 14 after UUO. Subsequently, the expression of Snail 1, which is a key molecule that triggers the process of EMT, became significantly elevated. Generally, when EMT occurs, tubular epithelial cells are transformed into myofibroblasts and produce interstitial components. EMT is characterized by a loss of epithelial morphology, which is accompanied by the downregulation of epithelial markers. Consistent with these, we found that renal vanin-1 protein decreased significantly on days 3 and 7, before trending to decrease on day 14 after UUO. Inversely, the mRNA expressions of TGF-β1 and Snail 1 increased in rats with UUO (Figure 3 and Figure 5) along with the histopathological spread of fibrosis (Figure 2). In this study, vanin-1 was not detectable in all injured tubules. It is considered that this phenomenon is partly due to decreased vanin-1 expression in cells with a loss of epithelial morphology.

Vanin-1 is attached to the cell membrane by GPI linkage [8]. The GPI anchor of vanin-1 can be cleaved, resulting in vanin-1 being secreted or released into the extracellular matrix environment. It has been reported that GPI-anchored proteins are easily released from the cell surface in response to various stimuli [22]. Importantly, in many epithelial cell lines, GPI-anchored proteins are located predominantly in the apical plasma membrane [23,24]. Indeed, we confirmed that vanin-1 was localized on the apical surface of injured renal tubules but not the basolateral surface by immunohistochemical analysis. Consistent with this, we found that serum vanin-1 exhibited no significant change in rats with UUO. Thus, in this model, the apical shedding of vanin-1 may have occurred, leading to the release of vanin-1 into the urine rather than serum. However, further studies are needed to clarify the molecular mechanisms responsible for the localization of vanin-1 under various stresses.

Tubulointerstitial fibrosis is the common final outcome of several progressive injuries, which leads to chronic renal failure. In the progression of renal disease, the tubulointerstitial influx of inflammatory cells, such as monocytes and T lymphocytes, has been observed [25] and these immune cells are recruited by leukocyte adhesion molecules and chemokines that are expressed in tubular epithelial cells. The persistence of inflammation in the kidney contributes to the development of tubulointerstitial fibrosis [26]. Therefore, it is important to detect renal tubular damage at an early stage and initiate appropriate therapeutic intervention. In the majority of previous UUO studies, voided urine was obtained as urinary samples using metabolic cages [27,28]. However, in rats with UUO, as the ureter was completely ligated, the voided urine was not from the ureter linked to the UUO kidney. In this study, we used the pelvic urine of UUO rats for urinalysis and this approach enabled us to follow the progression of renal injury of UUO. Therefore, urinary vanin-1 may be a potential biomarker of the onset of EMT during the progression of hydronephrosis.

## 4. Materials and Methods

### 4.1. Animals

All animal procedures were approved by the Committee of Animal Use and Care of Osaka Medical College (Permission number: 29095; Permission date: 29 May 2018), which was performed in accordance with the Guidelines for Animal Research. Adult male Sprague-Dawley rats were obtained at 7 weeks of age from Japan SLC (Shizuoka, Japan) and maintained under specific pathogen-free conditions with controlled temperature and humidity under a 12-h light-dark cycle. They had free access to water and a regular diet (CE-2; CLEA Japan, Tokyo, Japan) for 1 week before the experiment.

### 4.2. Experiments

After acclimatization for 1 week in cages, the rats were randomly divided into the sham-operated group (control group, *n* = 20) and UUO group (*n* = 20). The UUO surgery and the collection of samples were described previously. Briefly, under isoflurane anesthesia, the left ureter was exposed and ligated at two points. Control group rats underwent an identical surgical procedure, except for the ureteral ligation. The rats were allowed to recover from anesthesia, before being housed in standard rodent cages (3–4 rats in each cage) with ad libitum access to water and food until they were sacrificed. Voided urine from the sham-operated rats was collected over a 12-h period in a metabolic cage before being sacrificed. The voided urine from the rats with UUO were collected from the right reference kidney, while the urine in the pelvis from UUO rats was collected at the time of sacrifice. The rats were anesthetized with isoflurane to obtain blood and kidney tissues at 1, 3, 7 and 14 days after the operation.

### 4.3. Echographic Study

The echographic study was performed under isoflurane anesthesia using an echographic system (Vevo 1100 Imaging System, Transducer: MS250, FUJIFILM VisualSonics, Inc., Toronto, ON, Canada).

### 4.4. Laboratory Measurements

Urine and blood were centrifuged at 1000× *g* for 10 min, before the supernatant and serum samples were obtained. The creatinine concentration was measured by the Jaffe method using a commercial kit (Wako Pure Chemical Industries, Osaka, Japan), while vanin-1 was measured using an enzyme-linked immunosorbent assay (ELISA) kit (Uscn Life Science Inc., Wuhan, China).

### 4.5. Histological Analysis

Kidney tissues were fixed with Carnoy Solution (Muto Pure Chemicals Co., Ltd., Tokyo, Japan) for 24 h and embedded in paraffin, before being cut at a thickness of 4 μm. The sections were stained with periodic acid-Schiff (PAS) to evaluate tubular damage and with Masson’s trichrome for the assessment of interstitial fibrosis.

### 4.6. Immunohistochemical Analysis

The sections were deparaffinized with xylene, placed in a series of graded ethanol and washed with PBS. After this, they were pretreated with 10 mM citrate buffer (pH of 6.0) and autoclaved for 5 min at 120 °C for antigen retrieval. The sections were then soaked in 3% H_2_O_2_ in methanol for 5 min at room temperature to inhibit endogenous peroxidase activity. To suppress non-specific binding, the sections were incubated with protein-blocking solution (Dako, Tokyo, Japan) for 5 min. Following another PBS wash, the sections were incubated at 4 °C overnight with anti-vanin-1 antibody (Uscn Life Science Inc.) (1:50 dilution) or anti-4-HNE antibody (JaICA, Shizuoka, Japan) (1:100 dilution). After this, the slides were incubated with biotin-conjugated secondary antibody (LSAB 2 Kit/HRP; Dako, Japan) for 30 min after being washed in PBS. Thereafter, the sections were incubated with avidin-biotin-peroxidase complex (Dako) for 30 min, washed with PBS and subsequently incubated with 0.05% 3,3-diaminobenzidine. Finally, the slides were washed in running water, counterstained with hematoxylin and mounted with cover glasses.

### 4.7. Real-Time Quantitative PCR

RNA extraction and real-time quantitative PCR were performed as described previously. Specific sets of primers and TaqMan probes were obtained from Life Technologies. To control for variations in the amount of cDNA available for PCR in the different samples, the mRNA expression levels of the target sequences were normalized to the expression of an internal control, Glyceraldehyde-3-phosphate dehydrogenase (Gapdh). The GenBank accession numbers, assay ID and target exons were as follows: vanin-1, NM_001025623.1, Rn01537206_m1, and 6-7; Snail-1, NM_053805.1, Rn00441533_g1, and 1-2; TGF-β1, NM_021578.2, Rn00572010_m1, and 1-2; and Gapdh, NM_017008.3, Rn99999916_s1, and 1-1, respectively. Data were analyzed using the comparative threshold cycle method.

### 4.8. Measurement of Tissue Vanin-1 Protein 

Sample lysates were prepared from the kidneys of rats. After measuring protein concentrations using a BCA protein assay kit (Thermo scientific, Rockford, IL, USA), 25 μg of the total protein was loaded onto the microplate for ELISA for vanin-1 (Uscn Life Science Inc.).

### 4.9. Statistical Analyses

Data are presented as the means and standard error (SE). The differences between two groups were analyzed using the Mann-Whitney *U* test or the unpaired *t*-test. A value of *p* < 0.05 was considered to be significant. All statistical analyses were conducted with GraphPad Prism, version 4.03 (GraphPad Software, Inc., San Diego, CA, USA).

## 5. Conclusions

In this study, we showed the role of vanin-1 in the induction of tubular damage and subsequent renal fibrosis using rats with UUO. However, a causal association should be established by the use of pharmacological inhibitors of vanin-1 [29] or genetic models. In conclusion, vanin-1 that was released in the renal pelvic urine was detected in the UUO rats. In humans, obstructive nephropathy is always caused by incomplete obstruction, which is dissimilar to animal models. Therefore, clinical studies are needed to confirm urinary vanin-1 levels in patients with hydronephrosis.

## Abbreviations

AKI	acute kidney injury
EMT	epithelial-to-mesenchymal transition
Gapdh	glyceraldehyde-3-phosphate dehydrogenase
4-HNE	4-hydroxy-2-nonenal
UUO	unilateral ureteral obstruction
